# Copper Complexing Capacity and Trace Metal Content in Common and Balsamic Vinegars: Impact of Organic Matter

**DOI:** 10.3390/molecules25040861

**Published:** 2020-02-15

**Authors:** Sotirios Karavoltsos, Aikaterini Sakellari, Vassilia J. Sinanoglou, Panagiotis Zoumpoulakis, Marta Plavšić, Manos Dassenakis, Nick Kalogeropoulos

**Affiliations:** 1 National and Kapodistrian University of Athens, Department of Chemistry, Laboratory of Environmental Chemistry, Panepistimiopolis, Zografou, 15784 Athens, Greece; esakel@chem.uoa.gr (A.S.); edasenak@chem.uoa.gr (M.D.); 2 University of West Attica, Department of Food Science and Technology, Laboratory of Chemistry, Analysis & Design of Food Processes, Agiou Spiridonos 28, Egaleo 122 43, Greece; 3 Institute of Chemical Biology, National Hellenic Research Foundation, 48, Vas. Constantinou Ave., 11635 Athens, Greece; pzoump@eie.gr; 4 Ruđer Bošković Institute, Division for Marine and Environmental Research, P.O. Box 180, 10002 Zagreb, Croatia; plavsic@irb.hr; 5 Harokopio University of Athens, Department of Dietetics-Nutrition, School of Health Science and Education, El. Venizelou 70, Kallithea, 176 76 Athens, Greece; nickal@hua.gr

**Keywords:** copper complexation, organic ligands, trace metals, vinegars

## Abstract

Complex formation is among the mechanisms affecting metal bioaccessibility. Hence, the quantification of organic metal complexation in food items is of interest. Organic ligands in solutions of environmental and/or food origin function as buffering agents against small changes in dissolved metal concentrations, being able to maintain free metal ion concentrations below the toxicity threshold. Organic matter in vinegars consists of bioactive compounds, such as polyphenols, Maillard reaction endproducts, etc., capable of complexing metal ions. Furthermore, transition metal ions are considered as micronutrients essential for living organisms exerting a crucial role in metabolic processes. In this study, differential pulse anodic stripping voltammetry (DPASV), a sensitive electrochemical technique considered to be a powerful tool for the study of metal speciation, was applied for the first time in vinegar samples. The concentrations of Cu complexing ligands (*L*_T_) in 43 vinegars retailed in Greece varied between 0.05 and 52 μM, with the highest median concentration determined in balsamic vinegars (14 μM), compared to that of common vinegars (0.86 μM). In 21% of the vinegar samples examined, *L*_T_ values were exceeded by the corresponding total Cu concentrations, indicating the presence of free Cu ion and/or bound within labile inorganic/organic complexes. Red grape balsamic vinegars exhibited the highest density of Cu ligands per mass unit of organic matter compared to other foodstuffs such as herbal infusions, coffee brews, and beers. Among the 16 metals determined in vinegars, Pb is of particular importance from a toxicological point of view, whereas further investigation is required regarding potential Rb biomagnification.

## 1. Introduction

Vinegars are one of the few acidic condiments used as a flavoring agent, preservative and, in some countries, a healthy drink [1,2]. Wine vinegar is made from red or white wine and is the most widely used vinegar in the households of Mediterranean and Central Europe countries [3]. Vinegars are classified as “common” or “balsamic” according to their production from either wine or cooked grape must fermentation, respectively [4]. In the European Union, established limits for acidity are strictly specified. Thus, the acidity of wine vinegar (acetification obtained exclusively from wine) must be at least 6% *w*/*v* [5]. 

The fraction of metals which can actually be retained by the human body through vinegar consumption is related to the levels of metals in the harvest (primarily metals in grape juice and wine) as well as metal impurities due to manufacturing processes (chemicals added during production, materials used during transport, processing, or storage) [6] in addition to the bioavailability of metal species present in vinegar [7]. Metal bioaccessibility is affected by several natural mechanisms, among which complex formation is included. Additionally, due to hydrogen atom transfer and single-electron transfer, phenolic antioxidants protect against free radical formation by binding and/or inactivating metal ions [8,9,10]. Such a mechanism of antioxidant action is quite significant, despite the fact that the contribution of free radical scavenging or metal chelation to the overall antioxidant activity is still not fully specified [11]. 

It is well known that several toxic and/or biological effects of metals are attributed to their free or labile forms, which can actively interact with the binding sites of biological ligands [12,13]. The most labile form is the free dissolved metal ions, Me^x+^ (usually present as hydrated species), followed by dissolved inorganic metal ion pairs and complexes and organic complexes [14]. Differential pulse anodic stripping voltammetry (DPASV) is one of the few sensitive electrochemical techniques able to provide information on metal lability [15]. Typically, at low pH values, metal ions are present as the most labile species (as free ions or inorganic complexes) and are prone to different interactions [16].

Vinegars, depending on the raw material, origin, and type, are characterized by the presence of significant amounts of organic material composed by polyphenols, aminoacids, Maillard reaction endproducts, etc., prone to complexing metals and simultaneously representing the acidic matrix attributed to the presence of acetic acid. In such an environment, the study of metal complexation, for which available pertinent information is lacking, becomes challenging. 

Due to the fact that vinegars—being a dilute acetic acid solution—accelerate rusting, their content of trace elements turns out to be of particular interest. The reaction of acetate with chemical species (moieties) present in rust and grimes changes their makeup, hence resulting in their dissolution into water [17,18]. Although there are numerous published works on the concentrations of metals in wine, only a few refer to metal concentrations in vinegar [3,6,19,20,21].

Therefore, the objectives of the present study were (a) to evaluate the occurrence of ligands prone to complexing Cu ions, thereby “buffering” the concentrations of free metal ions, in cases where pertinent available data are lacking and (b) to determine 16 trace element concentrations of vinegars retailed in Greece. The interaction of Cu chemical speciation with bioactive compounds present in vinegars is also discussed. 

## 2. Results and Discussion

Organic carbon (OC) concentrations in the vinegar samples examined ranged from 17 to 278 mg L^−1^ (Table 1), with balsamic vinegars demonstrating a significantly higher, approximately 5-fold, median OC content (152 mg L^−1^) than common vinegars (30 mg L^−1^) (Mann–Whitney test, *p*<0.001). It is noteworthy that none of the common vinegars analyzed had an OC that exceeded 50 mg L^−1^, whereas in the balsamic vinegars, the lowest measured concentration was equal to 90 mg L^−1^ (Table 1).

The pH showed a slightly higher fluctuation in common vinegars (2.2–3.8; median 2.8) compared to balsamic vinegars (2.6–3.3; 3.0) (Table 1). Due to the acidity (low pH) of vinegars, the study of metal complexation becomes particularly interesting.

### 2.1. Copper Complexation

In environmental aqueous samples, copper toxicity is proportional to the concentration of inorganic (Cu^2+^, Cu^0^) copper species, rather than the sum of all copper species comprising total dissolved copper concentration [22,23]. The quantification of organic copper complexation has become increasingly important in natural waters and other natural systems since organic ligands effectively buffer the system against small changes in dissolved copper concentrations and are able to maintain the free Cu^2+^ concentration below the toxicity threshold [22]. In aqueous environmental settings dissolved organic carbon (DOC) occurs as a spectrum of different species, many of which may not have a specific chemical formula [24]. As such, the metal—binding capacity of different DOC fractions is highly variable and significant for environmental samples. 

**Figure 1:**
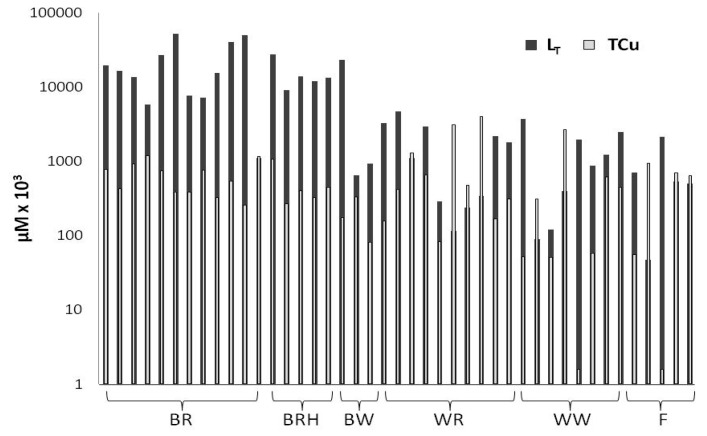
*L*_T_ and TCu concentrations (logarithmic scale) in vinegars (BR: balsamic red vinegar, *n* = 12; BRH: balsamic red vinegar with honey, *n* = 5; BW: balsamic white vinegar, *n* = 3; WR: wine red vinegar, *n* = 10; WW: wine white vinegar, *n* = 8; F: fruit vinegar, *n* = 5).

The *L*_T_ concentrations measured in vinegars are characterized by a significant variation among the different samples examined, with their values ranging from 0.05 μΜ in sample F2 to 52 μM in sample BR6 (Figure 1; Table 1). Balsamic vinegars demonstrated a considerably higher median *L*_T_ value (14 μM) compared to common vinegars (0.86 μM) (Mann–Whitney test, *p* < 0.001), with the highest median concentrations obtained for red grape balsamic vinegars (16 μM), followed by red grape balsamic vinegars with honey (13 μM) (Table 1). The lowest median *L*_T_ concentration was measured in fruit vinegars (0.54 μM). 

The values of log*K_app_*, expressing the stability of Cu–ligand complexes, ranged from 6.4 to 8.6 (Table 1). The identical median log*K_app_* values calculated herein for both balsamic (7.3) and common vinegars (7.3) (*p* > 0.05; Table 1) indicate the presence of ligands likely sharing similar chemical characteristics, despite the variation characterizing log*K_app_* values among the samples analyzed. A slightly higher median log*K_app_* value obtained for white grape balsamic vinegars (8.0) indicates the presence of rather different Cu ligands in the specific category. 

**Figure 2:**
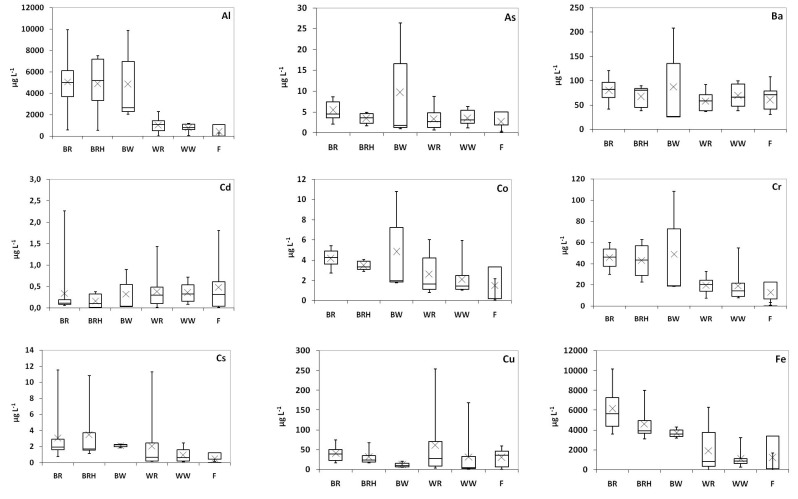

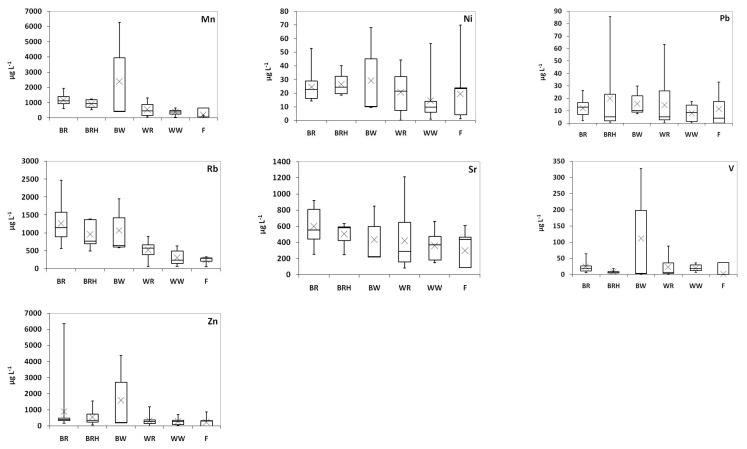
Plots of trace metals determined for all vinegar samples examined. The mean value is displayed with x. (BR: balsamic red vinegar; BRH: balsamic red vinegar with honey; BW: balsamic white vinegar; WR: wine red vinegar; WW: wine white vinegar; F: fruit vinegar).

Total Cu concentrations also varied significantly among the samples examined, ranging from <0.01 to 4.0 μM (Figure 2, Table 1), with 30% of white grape balsamic vinegars having total Cu concentrations exceeding 1.0 μM.

In 21% of the vinegar samples analyzed, *L*_T_ values were exceeded by total Cu concentrations, indicating the presence of labile Cu, either free or bound in weak complexes (Figure 1). This may be attributed to the high acidity of vinegar samples, with their pH lying between 2.2 and 3.8 (Table 1), and/or to their high Cu content. Almost 90% of the samples in which the presence of labile Cu was recorded were common vinegars, which are characterized by the lowest organic carbon concentrations. In common vinegars, high acidity combined with relatively low organic carbon concentrations seem to favor the presence of free/labile Cu ions.

The present results are considerably different from those obtained so far for samples of Greek herbal infusions [25], coffee brews [26], and Greek beers [27] in which the contained Cu was found to be fully complexed. It is quite peculiar that samples with such low pH values like vinegars exhibit *L*_T_, as it is known that the lower the pH of the sample, the lower the *L*_T_, since binding sites become protonated at lower pH levels and less prone to forming complexes. However, in the case of vinegars, it is obvious that compounds such as melanoidins (precursors of humic material), Maillard reaction endproducts, and phenolic compounds exist, capable of forming stable Cu complexes even at lower pHs. Similar features (i.e., high Cu complexing capacity) were observed for swamp water samples (Okefenokee swamps, Georgia, GA, USA) with a high content of humic material (even the color of the samples was light brown) and pH of 2.7 with *L*_T_ of 0.46 μM measured during the 1980s (personal communication with Dr. Marta Plavšić).

**Figure 3:**
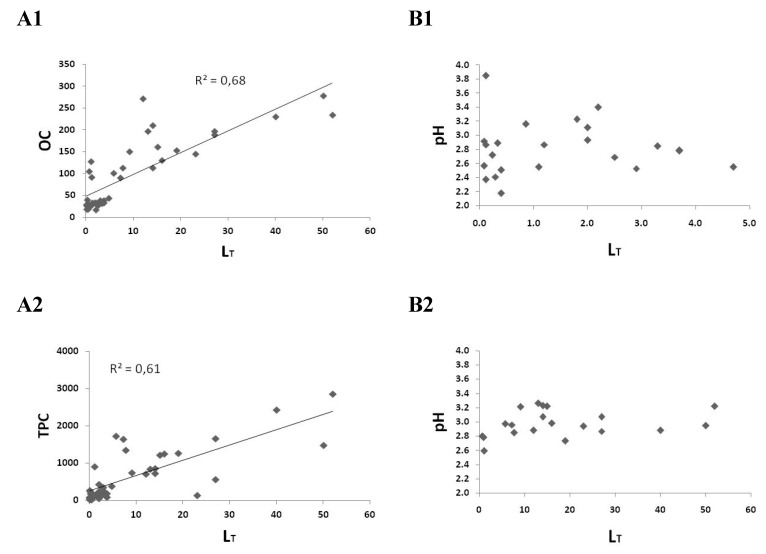
Correlation of *L*_T_ (μM) with organic carbon (OC) (mg L^−1^) (**A1**) and total phenolics (TPC) (mg GAE L^−1^) concentrations (**A2**) and correlation of *L*_T_ with pH values in common (**B1**) and balsamic vinegars (**B2**).

A Kruskal–Wallis H nonparametric test showed that there was a statistically significant difference in the median values of *L*_T_ (*p* < 0.001) and OC concentrations (*p* < 0.001), among the six subgroups into which the vinegar samples were classified. The aforementioned test also demonstrated that the differences in the median values among the different vinegar groups regarding pH (*p* = 0.152), Cu concentrations (*p* = 0.244), and log*K_app_* values (*p* = 0.891) were not statistically significant. It seems that *L*_T_ concentrations are related to the amount of organic matter present (Figure 3A1), whereas parameters such as acidity (Figure 3B1,B2) and nature of Cu ions ligands do not differentiate significantly among the vinegar categories examined.

Due to differences characterizing the organic matter content of vinegar samples, the normalization of *L*_T_ values in terms of organic carbon concentration provides information on the amount of Cu ligands per organic carbon mass unit. The density of Cu ligands per mass unit of organic matter (nmol Cu mg^−1^ C) was found higher in balsamic vinegars originating from red grapes (median concentration 122 nmol Cu mg^−1^ C in BR, 76 nmol Cu mg^−1^ C in BRH), compared to those calculated for the rest of the samples examined (45 nmol Cu mg^−1^ C in CR, 34 in CW, 21 in CF, 7.3 in BW) (Table 1). These values obtained for red grape balsamic vinegars are also higher compared to those of Greek herbal infusions, reaching 128 nmol Cu mg^−1^ C, coffee brews (31–59 nmol Cu mg^−1^ C) and Greek beers (0.91–7.0 nmol Cu mg^−1^ C) (Table S1; Supplemental Material). This comparison is feasible since in all aforementioned cases, *L*_T_ concentrations were determined by the same analytical method (DPASV), having the same “detection window”.

Copper complexes have revealed a significant performance in antioxidant studies [28], something confirmed by the positive correlation of *L*_T_ values with the total phenolic content (TPC; data in Sinanoglou et al. [29]) of vinegar samples (R^2^ = 0.61, *p* < 0.05) (Figure 3A2). Alonso et al. [30] have shown that in wine and other grape-derived products, a positive correlation can be established between the antioxidant activity and total phenolic content of samples, albeit not with specific compounds. Similarly, in vinegars, despite the correlation of complexing capacity of Cu ions with the total phenolic content of the samples, no corresponding correlations with specific categories of phenolic compounds (e.g., phenolic alcohols, phenolic acids, phenolic aldehyde; data in Sinanoglou et al. [29]) were observed.

The higher enrichment of the organic matter of red grape balsamic vinegars in Cu ligands is related with their production process. Contrary to white grape balsamic vinegars, the specific vinegars have undergone concentration and for their production, grape skins and seeds have been used. Similarly to red wine vinegars, red grape balsamic vinegars are expected to contain phenolic substances at levels even 10-fold higher than balsamic vinegars from white grapes. Polyphenols are the main phenolic compounds extracted from grapes during the winemaking process, initially obtained by fruit crushing and intensified through maceration and pumping-over processes carried out during fermentation [31]. Proanthocyanidins (condensed tannins) are among the most widespread polyphenol species in plants, often accumulating at large quantities in leaves, fruits, wood, or bark [32]. Most tannin molecules contain o-dihydroxyphenyl chelating functional groups, forming stable complexes with many metal ions [33,34].

### 2.2. Trace Elements

Sixteen trace elements, namely Al, As, Ba, Cd, Co, Cr, Cs, Cu, Fe, Mn, Ni, Pb, Rb, Sr, V, and Zn, were determined in the examined vinegar samples (Figure 2; Table S2). Median concentrations >1000 μg L^−1^ were determined for Fe and Al, between 10 and 1000 μg L^−1^ for Rb, Mn, Sr and Zn, between 10 and 100 μg L^−1^ for Cu, Cr, Ni, and V, and <10 μg L^−1^ for Pb, As, Co, Cs, and Cd. Arsenic, lead, and cadmium are particularly important environmental and industrial pollutants, currently ranked first, second, and seventh, respectively, in the 2017 Priority List of Hazardous Substances of the US Agency for Toxic Substances and Diseases Registry (http://www.atsdr.cdc.gov/SPL/), on the basis of their easy accessibility, toxicity, and potential for human exposure.

Arsenic concentrations in individual vinegar samples ranged from <0.05 μg L^−1^ in a fruit vinegar (apple) to 26 μg L^−1^ in a balsamic vinegar from white grapes, with median concentrations calculated equal to 2.6 and 4.2 μg L^−1^ in common and balsamic vinegars, respectively (Figure 2), without the difference being statistically significant (Mann–Whitney test; *p* = 0.095). In order to calculate the arsenic intake through the consumption of the studied vinegars, the PTDI (provisional tolerable daily intake) for As (126 μg per day for a 60 kg person) was withdrawn, since the BMRL0.5 value (lower limit on the benchmark dose for a 0.5% response) has been calculated at the same range, e.g., 180 μg per day (for a 60 kg person) as being responsible for causing lung cancer [35]. Considering a daily vinegar consumption equal to one tablespoon (15 mL), the maximum As daily intake from the studied vinegar was calculated to be equal to 0.40 μg, being significantly lower—almost up to 300 times—than the withdrawn PTDI limit.

The range of Pb concentrations measured herein extends from <0.05 μg L^−1^ in both an individual balsamic vinegar with fruits and several common vinegars to 86 μg L^−1^ in a balsamic red vinegar with honey. The second highest concentration (63 μg L^−1^) was detected in a balsamic sample from red grapes. The corresponding median Pb concentrations in common and balsamic vinegars were calculated at 6.0 and 9.8 μg L^−1^, respectively, without a statistically significant difference (*p* = 0.654). California State’s maximum level of Pb for human consumption (0.5 μg Pb per day), corresponding to Pb concentrations equal or higher than 34 μg L^−1^ [6], was exceeded in only 2 vinegar samples, representing 5% of those examined.

Cadmium concentrations ranged from 0.03 μg L^−1^ in both individual balsamic vinegars from red grapes with honey and common vinegars from red grapes and fruits (apple) to 2.3 μg L^−1^ in an aged balsamic vinegar from red grapes. Significant Cd concentrations were determined in common vinegars with fruits (apple) (1.8 μg L^−1^) and vinegars from red grapes (1.4 μg L^−1^). The median Cd concentration was higher in common (0.30 μg L^−1^) in comparison to balsamic vinegars (0.10 μg L^−1^) (*p* = 0.046). In any case, Cd concentrations determined in vinegar samples were significantly lower than the recommended PTDI (50 μg per day for a 60 kg person) [36], corresponding to a 3.3 mg L^−1^ concentration.

For the rest of trace elements examined, higher concentrations in balsamic compared to common vinegars were obtained for Al (median concentration 4657 μg L^−1^ in balsamic, 768 μg L^−1^ in common vinegar, *p* < 0.001), Cr (44 vs. 17 μg L^−1^, *p* < 0.001), Fe (4394 vs. 782 μg L^−1^, *p* < 0.001), Mn (1055 vs. 357 μg L^−1^, *p* < 0.001), Rb (1053 vs. 318 μg L^−1^, *p* < 0.001), Co (4.0 vs. 1.4 μg L^−1^, *p* = 0.004), Sr (555 vs. 329 μg L^−1^, *p* = 0.004), and Cs (1.9 vs. 0.60 μg L^−1^, *p* = 0.004). No statistically significant differences in median concentrations were observed between the two vinegar categories regarding Ba, Cu, V, Ni, and Zn (*p* > 0.05).

In order to evaluate any data trends and gain an overview of the relationships among the trace metals examined, principal component analysis (PCA) was performed individually for common and balsamic vinegar samples. In each factor analysis, metals which do not relate satisfactorily with the rest of the trace metals studied (measure sampling adequacy (MSA) values <0.5) were excluded. For the remaining ones, the value of the Kaiser–Meyer–Olkin (KMO) statistical criterion is equal to 0.86 and 0.85 for common and balsamic vinegars, respectively, and factor analysis is feasible. Two principal components (PCs) with eigenvalues >1 were extracted (PC1 explained up to 77% and 81% of the total variance for common and balsamic vinegars and PC2 explained up to 9% and 10%, respectively). In common vinegars, a statistically acceptable factor analysis is feasible for Al, Co, Fe, Mn, Ni, and Sr, whereas in balsamic vinegars, for As, Ba, Co, Mn, V, and Zn. According to the component plot obtained for balsamic vinegars (Figure S1), As, Ba, Co, Mn, and V are clustered well, whereas Zn appears to differentiate. Apparently, natural soil components constitute the main source of As, Ba, Co, Mn, and V, since the concentrations of trace metals such as As, Ba, Co, and V are not expected to be affected by processing and storage processes. Considering Zn, the anthropogenic impact, such as from the use of fertilizers, appears to be significant. It is noteworthy that in common vinegars, the clusters in PCA analysis (Figure S1) are more scattered compared to those in balsamic vinegars, demonstrating weaker relationships among the trace metals examined. In common vinegars in which neither the raw material quality nor the technological producing procedures are controlled as effectively as in balsamic vinegars, there are multiple sources of trace metals which are not common to all samples. No satisfactory discrimination was achieved for the two vinegar categories by the factor analysis score plots of trace metals. 

In order to obtain additional information on metal concentration levels and their potential magnification in vinegars, indicative enrichment factors (IEFs) were calculated for individual elements in terms of the average elemental composition of the upper continental crust, since the main raw materials in vinegar production are grape juice and/or wine, where the trace metals that enter originate from the soil during vine growth. The enrichment factors calculated herein are only indicative, since metals differ in their reactivity and this influences their availability for uptake from the soil by plants (e.g., vineyards) and transfer into the food chain [37,38]. For each one of the examined trace metals, Fe was used as the reference element considering the upper continental crustal composition given by Rudnick and Gao [39]. The indicative enrichment factor (IEF) of an element E is defined according to Equation (1):

IEF= (E/R)_vinegar_/(E/R)_crust_, (1)

where E and R represent the concentrations of examined and reference element, respectively. Operationally, if IEF approaches 1, no significant alteration of the examined element from the relative composition of the earth’s crust exists, whereas high IEFs indicate significant alteration from local variation in soil composition and possible magnification in vinegar samples. IEF values close to 100 were determined for Rb, Zn, and Cd; close to 50 for As; and between 10 and 25 for Cu, Mn, Pb, and Cs; whereas for the rest of the trace elements studied, Efs<10 were calculated. Zn, Cu, and Mn are present as necessary trace elements in fertilizers [40,41] that are applied in viniculture. Copper, either alone or combined with sulfur, is extensively applied as a pesticide in vineyards [42]. Both Cd and Rb are present in phosphate fertilizers, originating from parent raw materials [43,44]. Cultivation practices applied in viniculture, such as the use of fertilizers and plant protection products, potentially contribute to the trace metal content of vinegars, for which significant IEFs were calculated, albeit that it was not possible to evaluate the contribution from the use of either tools or storage media. Despite the relatively high IEFs obtained for Zn, its concentrations measured in vinegar samples were significantly lower compared to the recommended PMTDI (provisional maximum tolerable daily intake) (18–60 mg per day for a 60 kg person) [45], corresponding to a concentration ranging from 1200 to 4000 mg L^−1^. Regarding Rb, for which high IEFs were also obtained, it demonstrates transport mechanisms in plant roots similar to those of K, which constitutes an essential element for plants [46]. Recently, at a coastal area of Israel in eastern Mediterranean Sea, elevated Rb concentrations were reported in soils, soil solutions, rainwater through fall water and plant litter leachates, with sea aerosols constituting a major input [47]. Similarly, Campbell et al. [48] proposed that Rb should be included as a metal that is consistently biomagnified in diverse food webs and should hence be considered in multi-element biomagnification studies.

Table 2 shows a comparison of the concentrations of trace metals examined in the present study with those reported in literature for vinegars produced and/or retailed in different places. Trace metals levels lying in the lower range of the concentrations reported in literature were determined in the present work. A high variation characterizes the concentrations of specific metals such as Cd, Cr, Cu, Fe, Ni, Pb, and Zn reported in the different studies, possibly due to a contamination of the raw material and/or to technological manipulations constituting potential sources of these metals in vinegars, with the latter appearing to be more important. Similarly, Ndung’u et al. [6], studying the Pb isotopic fingerprints in vinegars, reported that the main Pb sources are anthropogenic, with chemicals added during production and/or material used during transport, processing, or storage being significant. Table vinegars typically contain no less than 4% per weight acetic acid, which undergoes the typical chemical reactions of carboxylic acids, being corrosive against mild steel and accelerating the dissociation of metals, including Fe, Mg, and Zn [17].

## 3. Materials and Methods

### 3.1. Sampling

Forty-three (twenty-three common and twenty balsamic) vinegars were purchased from different producers (Table 1). The vinegar samples were furthermore categorized in vinegars produced from red (WR; *n* =10) and white wine (WW; *n* =8), fruit vinegars (F; *n* =3), balsamic vinegars from red grapes (BR; *n* =12), balsamic vinegars from red grapes with honey (BRH; *n* =5), and balsamic vinegars from white grapes (BW; *n* =3) (Table 1).

### 3.2. Electrochemical Measurements

#### 3.2.1. Sample Preparation and Equipment

For the determination of *L*_T_, 5 mL of vinegar samples were 10-fold diluted with Milli-Q water, 18.2 MΩ cm (Millipore, Bedford, MA, USA). Following addition of 5 drops of 3 M NaCl, samples were immediately subjected to copper complexing capacity (*L*_T_) and apparent stability constant (*K_app_*) determinations.

Electrochemical measurements were carried out using a μAutolab type III (Eco-Chemie, Utrecht, The Netherlands) instrument connected to a three-electrode cell (663 VA Stand, Metrohm, Herisau, Switzerland) with a static mercury drop electrode (SMDE) as the working electrode. The reference electrode was an Ag/AgCl (3 M KCl). A carbon rod electrode served as the auxiliary electrode.

#### 3.2.2. Copper Complexing Capacity (*L*_T_)

Differential pulse anodic stripping voltammetry (DPASV) was used for complexing capacity determinations [15]. The imposed experimental conditions on the electrode system were modulation time 0.04s, interval time 0.31 s, modulation amplitude 25 mV, and step potential 5 mV. The measurements were performed by direct titrations of the samples, to which the supporting electrolyte (NaCl) was added in the voltammetric cell with the standard addition of copper ions. The equilibration time of 15 min between the addition of copper ion and the measurement was proven adequate for attaining equilibrium in the sample. During the accumulation period in the cell a potential of −0.6V vs. Ag/AgCl reference electrode was applied, and copper ions were reduced on the electrode in the form of amalgam. In the oxidation step (stripping step) which follows, copper is oxidized from the amalgam, giving rise to a voltammetric peak, the height (current) of which is then used for the calculation of *L*_T_. The sensitivity of the method and the total amount of copper ions present were determined in the same way in the acidified (pH=2) sample solution, using a standard additions titration procedure. 

**Figure 4:**
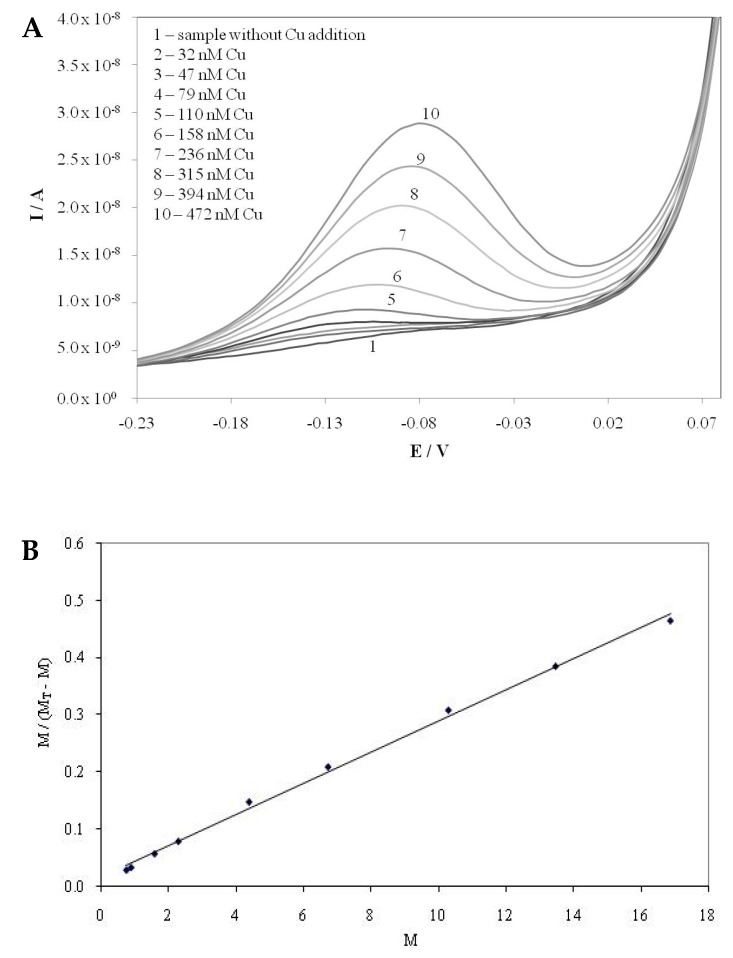
DPASV voltammograms of Cu ion titrations (**A**) and Ruzic–van den Berg plot (**B**) for the balsamic vinegar sample BR5.

When all ligands capable of complexing copper ions in the sample are saturated with the added copper ions, the slopes of the titration lines at natural pH and acidified samples become parallel and the approximate *L*_T_ value is calculated by extrapolation of these titration lines to the *x*-axis. Exact values of copper complexing capacity (*L*_T_) as well as of corresponding apparent stability constants (*K_app_*) were calculated by applying the linear transformation plot through linearly transforming titration data, assuming 1:1 metal to ligand complexation [58,59]. The equation used for calculation is [Cu]/[CuL] = [Cu]/*L*_T_ + 1/*KL*_T_, where Cu is the copper ion detected by anodic stripping voltammetry, CuL is the copper ion bound in a complex with ligand L, *L*_T_ is the total concentration of binding ligands (complexing capacity), and *K* is the apparent stability constant. The plot of [Cu]/[*L*_T_] vs. [Cu] yields a straight line with a slope of 1/*L*_T_ and an intercept of 1/*KL*_T_ (Figure 4). The calculated repeatability (*n* =5) was found lower than 10%.

### 3.3. Trace Metals

Vinegar samples were digested with HNO_3_ 65% supra pure (Merck, Darmstadt, Germany) with subsequent addition of H_2_O_2_ 30% according to Sakellari et al. [27]. For the determination of Al, As, Ba, Cd, Co, Cr, Cs, Cu, Fe, Mn, Ni, Pb, Rb, Sr, V, and Zn, the digested samples were analyzed through inductively coupled plasma mass spectrometry (ICP–MS) by a Thermo Scientific ICAP Qc (Waltham, MA, USA). Measurements were carried out in a single-collision cell mode, with kinetic energy discrimination (KED) using pure He. Matrix induced signal suppressions and instrumental drift were corrected by internal standardization (^45^Sc, ^103^Rh). Measurements were performed in triplicate.

Precision repeatability (*n* = 5) was <15% for all trace metals. The LODs (in μg L^−1^) were equal to 0.02 for Co and Cs; 0.03 for Cd, 0.05 for As, Pb, and V; 0.10 for Ba, Cr, Rb, and Sr; 0.20 for Mn, 0.40 for Cu and Ni; 1.0 for Zn; 2.0 for Al; and 3.0 for Fe [60]. For statistical calculations, values below the LOD were assigned the limit of detection divided by 2. 

The quality assurance was provided by recovery tests through the analysis of metal spiked samples. Recovery efficiency for spiked sample analysis was 77.8% for Zn, 82.1% for Cr, 88.2% for Fe, 89.2% for Pb, 89.8% for Ni, 90.2% for V, 93.6%for Sr, 95.8% for Ba, 97.1% for Mn, 97.2% for Cs, 97.5% for Cd, 98.3%for Cu, 104.2% for Al, and 107.7% for As. The certified reference materials (CRM) ERM^®^-CD281 (rye grass) and BCR No 279 (trace elements in sea lettuce *Ulva lactuca*) were analyzed and the recoveries for As, Cd, Cr, Cu, Mn, Ni, Pb, and Zn were in the range ±20%. 

### 3.4. Organic Carbon (OC) and pH

Total organic carbon (OC) was determined by high temperature catalytic oxidation employing a TOC-5000A Shimadzu analyzer (Shimadzu Scientific Instruments, Columbia, MD, USA). The precision was estimated as the standard deviation between injections and was less than 2% of the mean.

The pH of the samples was measured with a Jenway 3310 pH-meter (Jenway LTD, Felsted, Dunmow, Essex, UK).

### 3.5. Statistical Analysis

The Kolmogorov–Smirnov and Shapiro–Wilk tests were used to assess the normality of data. Both tests gave *p* values less than 0.05 and the null hypothesis (that the data are normally distributed) was rejected. Therefore, the non-parametric tests Mann–Whitney U and Kruskal–Wallis were used to statistically compare values between two groups and more than two groups, respectively. Principal component analysis (PCA) was used in order to evaluate any data trends and gain an overview of the relationships among the trace metals examined. PASW Statistics for Windows, Version 24.0, was used as a statistical package (SPSS Inc, Chicago, IL, USA).

## 4. Conclusions

Metal chelation comprises, together with free radical scavenging and reducing capacity, the modes of antioxidant action exerted by plant phenolics. The organic matter in vinegars includes ligands such as polyphenols capable of complexing metal ions. pH is a factor which strongly influences metal complexation with organics. Due to the low acidity of vinegars, the study of metal complexation is of particular interest. 

Copper complexing capacity (*L*_T_) was determined for the first time in 43 vinegars by differential pulse anodic stripping voltammetry (DPASV). Copper ions were not efficiently “buffered” by the organic matter, since in 21% of the samples, total Cu concentrations exceeded the corresponding *L_T_* values. The density of Cu ligands per mass unit of organic matter was found to be higher in balsamic vinegars originating from red grapes compared to those of herbal infusions, coffee brews, and beersas determined in previous studies. A positive correlation of *L*_T_ values with the total phenolic content (TPC) of vinegar samples (R^2^ = 0.61, *p* < 0.05) was found.

Anthropogenic sources, such as the application of fertilizers, and plant protection practices in combination with technological manipulations (e.g., material used during transport, processing, or storage) seem to significantly contribute to the observed concentrations of Rb, Zn, Cd, Cu, Mn, Pb, and Cs in vinegars.

## Figures and Tables

**Table 1:** Copper complexation parameters (*L*_T_, log*K_app_*), total Cu concentration, organic carbon content, pH, and *L*_T_/TOC values in different categories of vinegars.

Sample No	Vinegars	pH	*L*_T_ (μM)	log*K_app_*	Cu (μM)	OC (mg L^−1^)	*L*_T_/OC (nmol Cu mg^−1^ C)
**BR (Red Grape Balsamic Vinegars)**							
BR1	Balsamic red	2.7	19	8.6	0.77	154	126
BR2	Balsamic red	3.0	16	7.2	0.43	130	126
BR3	Balsamic red	3.1	14	7.4	0.92	114	118
BR4	Balsamic red	3.0	5.7	7.6	1.2	102	56
BR5	Balsamic red	3.1	27	6.9	0.74	197	135
BR6	Balsamic red	3.2	52	6.6	0.38	235	222
BR7	Balsamic red	2.8	7.7	7.8	0.38	113	68
BR8	Balsamic red	3.0	7.2	7.9	0.75	90	80
BR9	Balsamic red	3.2	15	7.1	0.32	162	96
BR10	Balsamic red	2.9	40	6.4	0.54	230	174
BR11	Balsamic red bio	3.0	50	6.8	0.26	278	178
BR12	Balsamic red bio	2.6	1.1	7.8	1.2	92	12
mean ± SD		3.0 ± 0.2	21 ± 17	7.3 ± 0.6	0.66 ± 0.33	158 ± 63	116 ± 58
median		3.0	16	7.3	0.64	142	122
**BRH (Red Grape Balsamic Vinegars with Honey)**							
BRH1	Balsamic red honey	2.9	27	7.8	1.1	189	144
BRH2	Balsamic red honey	3.2	9.1	7.8	0.27	151	60
BRH3	Balsamic red honey	3.2	14	7.2	0.40	211	66
BRH4	Balsamic red honey	2.9	12	7.2	0.32	273	44
BRH5	Balsamic red honey	3.3	13	6.7	0.44	197	68
mean ± SD		3.1 ± 0.2	15 ± 7	7.4 ± 0.6	0.51 ± 0.34	204 ± 44	76 ± 39
median		3.2	13	7.2	0.40	197	66
**BW (White Grape Balsamic Vinegars)**							
BW1	Balsamic white	2.9	23	7.0	0.17	145	161
BW2	Balsamic white	2.8	0.64	8.1	0.33	106	6.1
BW3	Balsamic white	2.8	0.94	8.0	0.08	128	7.3
mean ± SD		2.8 ± 0.1	8.2 ± 12.8	7.7 ± 0.6	0.19 ± 0.13	126 ± 20	58 ± 89
median		2.8	0.94	8.0	0.17	128	7.3
**WR (Wine Red Vinegars)**							
WR1	Wine red	2.8	3.3	7.8	0.16	32	102
WR2	Wine red	2.6	4.7	7.2	0.42	44	106
WR3	Wine red	2.6	1.1	7.3	1.3	30	36
WR4	Wine red	2.5	2.9	6.4	0.65	40	74
WR5	Wine red	2.4	0.29	7.3	0.08	28	10
WR6	Wine red	2.9	0.12	7.1	3.1	27	4.2
WR7	Wine red	2.7	0.24	7.5	0.47	41	5.8
WR8	Wine red	2.9	0.34	8.4	4.0	31	11
WR9	Wine red	3.4	2.2	7.6	0.16	30	73
WR10	Wine red	3.2	1.8	8.0	0.31	34	53
mean ± SD		2.8 ± 0.3	1.7 ± 1.6	7.5 ± 0.6	1.1 ± 1.4	33.7 ± 5.8	48 ± 40
medium		2.8	1.4	7.4	0.44	31.6	45
**WW (Wine White Vinegars)**							
WW1	Wine white	2.8	3.7	7.7	0.05	39	95
WW2	Wine white	2.6	0.09	7.2	0.31	30	3.0
WW3	Wine white	2.4	0.12	7.1	0.05	28	4.3
WW4	Wine white	2.5	0.40	7.2	2.7	28	14
WW5	Wine white	3.1	2.0	6.7	<0.01	17	114
WW6	Wine white	3.2	0.86	8.5	0.06	26	33
WW7	Wine white with rosemary and thyme	2.9	1.2	7.1	0.61	34	36
WW8	Champagne wine	2.7	2.5	7.8	0.44	28	88
mean ± SD		2.8 ± 0.3	1.4 ± 1.3	7.4 ± 0.6	0.53 ± 0.90	28.8 ± 6.3	48 ± 44
median		2.7	1.0	7.2	0.18	28.1	34
**F (Fruit Vinegars)**							
F1	Apple	2.8	3.7	7.9	0.06	34	21
F2	Apple	2.9	0.09	7.2	0.94	29	1.6
F3	Apple	3.8	0.12	7.0	<0.01	19	113
F4	Pomegranate	2.2	0.40	7.4	0.70	19	28
F5	Sea Buckthorn	2.9	2.0	7.1	0.63	33	15
mean ± SD		2.9 ± 0.6	0.78 ± 0.78	7.3 ± 0.4	0.47 ± 0.41	26.7 ± 7.4	36 ± 44
median		2.9	0.54	7.2	0.63	29.1	21

**Table 2:** Concentrations of trace metals in different types of vinegars reported in literature. Countries refer to place of retail.

	Al (mg L^−1^)	As (μg L^−1^)	Ba (μg L^−1^)	Cd (μg L^−1^)	Co (μg L^−1^)	Cr (μg L^−1^)	Cu (μg L^−1^)	Fe (mg L^−1^)	Mn (mg L^−1^)	Ni (μg L^−1^)	Pb (μg L^−1^)	Sr (mg L^−1^)	V (μg L^−1^)	Zn (mg L^−1^)
**Wine vinegars**														
Spain [3]	0.85-7.11	<20	40–164	<1	3–77	17–2090	17–8580	1.18–19.0	0.049–1.66	2–903	35–2200	0.196–1.83	11–147	0.040–6.12
Spain [49]		n.d.–23					20–6170	1.24–75.8	0.15–5.44					0.06–8.56
Spain [50]							320 ± 130							0.72 ± 0.11
Spain [51]		136					1350 ± 40	8.44 ± 0.07			550 ± 50			7.86 ± 0.51
Brazil [20]	0.12–2.98		20–400				10–50		0.04–10.0					<0.01–2.0
Brazil [52]				0.02–0.89										
Canary islands [53,54]				12–245			1440 ± 380	2.08 ± 1.09		48–205	360–1280			1.63 ± 0.28
Turkey [55]							130 ± 19	4.25 ± 1.95	0.78 ± 0.43	50 ± 27	20 ± 24			0.10 ± 0.019
Turkey [56]	0.55–3.07				n.d.–620	2950–3950	10–130	0.38–5.12	0.05–2.49		10–150			0.16–2.07
Iran [57]				n.d. –59.3			12.8–584				3.32–167			0.03–1.9
California, USA [19]											36–50			
California, USA [6]											7.4–590			
Greece *	<0.002–2.3	0.66–8.7	37–100	<0.030–1.4	0.78–6.0	7.5–55	<0.40–254	<0.003–6.3	0.04–1.3	<0.40–56	<0.050–63	0.08–1.2	0.31–88	0.01–1.2
Balsamic vinegars														
Turkey [55]				20 ± 5			420 ± 153	6.94 ± 1.50	1.31 ± 0.18	30 ± 19	20 ± 25			0.36 ± 0.162
Turkey [56]	0.56–2.29				n.d.260	1980–2090	10–30	2.51–7.01	0.02–0.14		10			0.26–0.79
California, USA [19]											15–307			
California, USA [6]											14–720			
Greece *	0.56–10	0.93–26	27–208	<0.030–2.3	1.8–11	19–109	5.2–75	3.1–10	0.43–6.3	9.6–68	<0.050–86	0.23–0.92	1.9–328	0.06–6.4
**Fruit vinegars**														
CanaryIslands [52,53]				12–37			570 ± 140	1.14 ± 0.45		48–201	350–550			0.25 ± 0.04
Turkey [55]				10 ± 4			30 ± 32	1.31 ± 0.58	0.18 ± 0.13	10 ± 13	10 ± 8			
Turkey [56]	0.32–5.19					1970–2330	10–50	0.27–6.84	0.03–1.51		10–80			0.29–2.42
Iran [57]				n.d. –78			32–1129				4.4–253			0.05–3.7
California, USA [6]											3.7–7.4			
Greece *	<0.002–1.4	<0.050–5.4	31–108	<0.030–1.8	0.08–5.5	0.88–26	<0.40–60	<0.003–5.1	<0.01–0.83	1.3–70	<0.050–33	0.09–0.61	<0.050–6.2	<0.01–0.88

* Present study

## Supplementary Materials

The following are available online at https://www.mdpi.com/1420-3049/25/4/861/s1, Table S1: Normalized values of *L*_T_ in terms of organic carbon concentration in a variety of beverages, infusions and food additives, Table S2: Trace metals content (μg L^−1^) of vinegars studied (mean value ± standard deviation), Figure S1: PCA analysis of trace metals in common (A) and balsamic (B) vinegars.
