# Supplementary material for: Copper Complexing Capacity and Trace Metal Content in Common and Balsamic Vinegars: Impact of Organic Matter
Source: Molecules. 2020 Feb 15;25(4):861. doi: 10.3390/molecules25040861 (PMC7070403; doi:10.3390/molecules25040861)
Supplement: Supplementary file 1 [file molecules-25-00861-s001.pdf]

Table S1. Normalized values of L_T in terms of organic carbon concentration in a variety of beverages, infusions and food additives.

Category	L_T /OC (nmol Cu mg^{-1} C)
**Herbal infusions[25]**	16 - 128 (56)
**Beers[27]**	0.9 - 7.0 (2.8)
**Coffee brews[26]**	29 - 70 (43)
**Vinegars*** **common**	2 -114 (33)
**Vinegars** **balsamic**	6 - 222 (88)

* present study

Table S2. Trace metals content (μg L^{-1}) of vinegars studied (mean value ± standard deviation)

	**Balsamic vinegars**			**Common vinegars**		
	**BR**	**BRH**	**BW**	**WR**	**WW**	**F**
Al	5079±2381	4880±2802	4857±4361	1044±713	797±379	426±562
As	5.4±2.3	3.4±1.4	9.7±14	3.4±2.5	3.6±1.8	2.6±1.9
Ba	80±26	68±23	87±105	58±19	70±24	61±30
Cd	0.34±0.62	0.16±0.17	0.32±0.50	0.38±0.42	0.36±0.23	0.48±0.75
Co	4.2±1.0	3.5±0.5	4.8±5.2	2.6±2.0	2.1±1.7	1.5±2.3
Cr	46±10	43±17	49±52	20±8	19±16	13±9
Cs	3.1±3.0	3.5±4.1	2.1±0.3	2.1±3.4	0.93±0.86	0.52±0.22
Cu	41±20	32±21	12±8	62±88	32±57	31±25
Fe	6136±2244	4620±1929	3712±554	1928±2401	1097±909	1220±2174
Mn	1188±423	929±293	2384±3372	540±439	371±184	223±342
Ni	25±11	27±9	29±34	21±15	14±18	20±28
Pb	12±7	20±37	16±12	15±20	8.2±6.9	12±14
Rb	1266±539	961±404	1064±770	520±264	309±215	244±108
Sr	600±219	505±156	435±359	419±351	360±178	297±226
V	24±18	7.8±5.8	111±188	22±31	20±11	21±2
Zn	901±1723	565±578	1605±2408	336±332	291±222	236±370

BR: Red grape balsamic vinegars (n=12); BRH: Red grape balsamic vinegars with honey (n=5); BW: White grape balsamic vinegars (n=3); WR: Red grape vinegars (n=10); WW: White grape vinegars (n=8); F: Fruit vinegars (n=5)

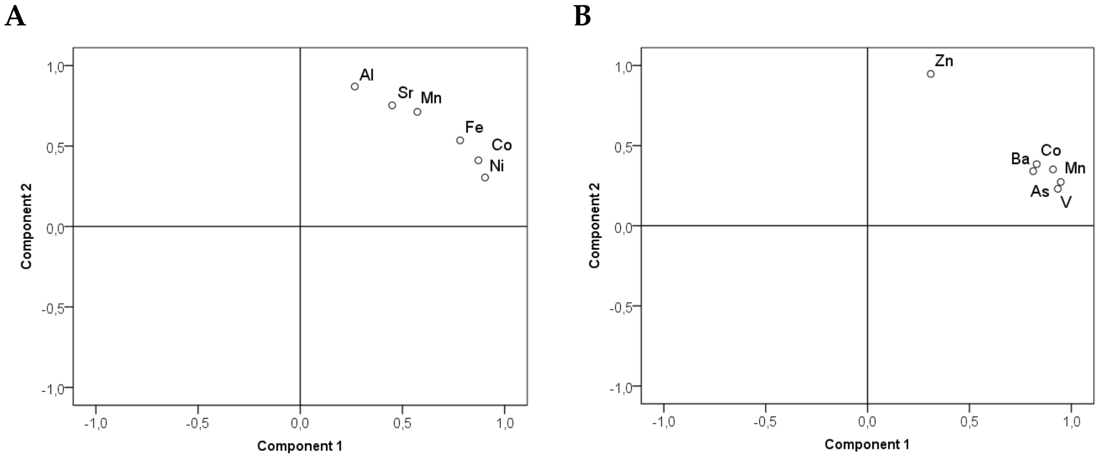

Figure S1. PCA analysis of trace metals in common (A) and balsamic (B) vinegars.
